# The importance of physician engagement in medical research

**DOI:** 10.3389/fmed.2025.1537023

**Published:** 2025-02-27

**Authors:** Oscar Gonzalez-Perez, Cesar Ramos-Remus

**Affiliations:** ^1^Laboratorio de Neurociencias, Facultad de Psicologia, Universidad de Colima, Colima, Mexico; ^2^Unidad de Investigacion en Enfermedades Crónico Degenerativas, Guadalajara, Mexico

**Keywords:** evidence-based medicine, clinical research, continuing medical education, healthcare quality, health policy

## 1 Introduction

In contemporary medicine, advancement and progress are inevitably linked to the rigor of empirical discovery and the strength of scientific validation. Therefore, the foundation for translating such scientific advances into clinical use lies in healthcare professionals. Thus, integrating clinical practice and teaching with scientific research is essential and has profound implications for healthcare quality and societal advancement (1). Also, to integrate patient-centered medicine (PCM) and patient-oriented research into clinical practice, it is essential to address key ethical, cultural, and methodological challenges (2–4). Ethically, we must move toward a model that values patient autonomy, establishing frameworks that enhance informed consent and shared decision-making. This would empower patients to engage actively in their healthcare. Culturally, initiatives should promote collaboration between patients and healthcare providers, ensuring that patient preferences and values align with clinical decisions. Methodologically, research needs to focus on individual patient experiences, combining quantitative and qualitative approaches to capture their complexities. Utilizing electronic medical records and predictive models will support more personalized care. By assembling these interconnected challenges, we can align clinical research with the diverse needs of patients, improving medical practice and clinical outcomes. Importantly, before conducting either observational studies (focused on understanding patient experiences and contexts without interference) or interventional studies (aimed at testing the effectiveness of specific interventions), all physicians must be aware of and follow globally accepted regulatory rules, including the good clinical practice guidelines (GCP), the Declaration of Helsinki, the international guidelines for biomedical research involving human subjects, as well as institutional guidelines issued in their home countries (5).

These issues highlight the importance of physicians proactively engaging in research endeavors, elucidating the multifaceted consequences of their involvement or lack thereof. Evidence-based medical practice and translational research form the bedrock of translating scientific discoveries to clinical applications and fast-tracking the process of converting research findings into tangible benefits to improve public health (6–10). Thus, it may hold little significance for most practitioners without an effective mechanism for translating and disseminating this knowledge. In this short commentary, we expose a general viewpoint about some benefits and risks associated with this practice and highlight the critical importance of fostering a culture of research-oriented clinical practice that aids in designing and promoting public policies to accelerate medical knowledge and learning and improve patient care.

## 2 The role of physicians in research

Physicians' involvement in medical research is supported by at least five basic principles that are mainly guided by motivational factors. These personal factors can be motivated by the desire to bridge clinical practice with research, enhance clinical expertise, advance medical knowledge, promote professional development, and influence health policy and practice guidelines. Below, we tried to rationalize each of them.

### 2.1 Bridging clinical practice with research

Healthcare professionals are well-versed in clinical matters, patient needs, and the real-life implications of medical interventions. Research participation helps them contextualize scientific knowledge into practice by formulating critical clinical questions, refining hypotheses, and developing solutions to improve patient outcomes. Hence, clinical expertise integrated with scientific curiosity bridges the gap between theory and practice.

### 2.2 Improvement of clinical skills

Research engagement also helps healthcare professionals to get crucial skills like critical thinking, data analysis, and evidence-based decision-making. This enhances their clinical judgment and enables them to provide medical assistance based on the latest scientific evidence. Thus, the commitment to research not only advances their professional careers but also helps elevate healthcare standards (11). Additionally, continuous learning through research keeps healthcare professionals at the forefront of medical advancements, improving the quality of care they deliver.

### 2.3 Advancing medical knowledge

The interaction with diverse patient populations provides healthcare professionals with valuable insights for clinical research that enhances their understanding of disease mechanisms, clinical responses, and long-term therapy outcomes. In other words, this helps expand medical knowledge and foster innovations in diagnosis, treatment, and prevention. Nevertheless, disseminating this knowledge is an indispensable step to promote the continuous development of medicine and improve the overall quality of healthcare.

### 2.4 Professional development

Healthcare professionals engaged in research are more likely to pursue additional training, develop specialized expertise, and contribute to academic medicine. The active engagement in research will allow healthcare professionals to enhance their understanding of medical science and develop extensive networks with peers. Such interactions allow the sharing of knowledge and establish mentorship relationships that can further guide future research and clinical activities.

### 2.5 Shaping health policy and practice guidelines

One of the main contributions of clinical researchers is the design, writing, and adjustment of health policies and guidelines on clinical practice. Thus, the findings of their studies provide the basis for evidence-based recommendations that shape standards of care both locally and internationally. These clinical guidelines are kept abreast of current research to ensure that patient care is continuously optimized to the best evidence available, thus improving health outcomes and furthering the science of medicine worldwide.

## 3 Consequences of physicians engaging in research

Participating in research has multifaceted consequences for healthcare professionals, and understanding these would help healthcare systems strategically support medical research engagement toward improved clinical outcomes and professional satisfaction (12, 13). Some identified positive outcomes are:

### 3.1 Improved patient care

Healthcare professionals in research are also more able to apply current scientific breakthroughs to the clinical arena. This ascertains that patients receive superior treatments and advance with the science of medicine. Hence, research-oriented clinicians can individualize care, optimize therapeutic regimens, and apply innovative approaches, thus improving patient outcomes.

### 3.2 Innovation and breakthroughs

Research developed by physicians fuels medical innovation due to clinical questions emanating from practical realities. Very valuable clinical insights are foregone without clinicians' involvement, thus retarding new diagnostic tool development, therapeutic interventions, and preventive measures. Missed opportunities for innovation associated with these benefits will slow medical progress and limit improvements in patient outcomes.

### 3.3 Enhanced collaboration and networking

Healthcare professionals engaged in research are often exposed to scientists, researchers, and other clinicians from diverse fields. This interaction and feedback accelerate knowledge sharing, encourage innovations, and facilitate comprehensive solutions to complex medical issues.

### 3.4 Funding and resources

Healthcare professionals doing research are also more likely to get grants and funding from government, non-government, and private organizations. Such financial support enables them to build state-of-the-art research facilities and develop newer technologies with high-class clinical trials. Thus, the more funding increases, the higher the chances of hiring and retaining renowned researchers, which improves the institution's research performance.

## 4 Consequences of physicians not engaging in research

In high-income countries, undesirable outcomes may occur when physicians fail to engage in research. However, most of these consequences are more evident in economically challenged countries (14–19). Some of the identifiable undesirable outcomes include (A) Hindrance in the advancement of medical knowledge: Without active participation, the medical community may struggle to generate new insights and responses to health challenges. (B) Decreased quality of patient care: Physicians lacking research involvement may miss out on the latest developments, leading to less effective care. (C) Limited professional growth: Not engaging in research can restrict physicians from gaining new skills and achieving academic recognition, affecting their career trajectory. (D) Missed opportunities for innovation: Physician insights are valuable for clinical advancements that may go unexplored, slowing the progress of medical innovations. (E) Reduced impact on health policy: Clinicians engaged in research play a crucial role in shaping health policy and clinical practice guidelines. Without their contributions, evidence-based recommendations may lack the practical insights and clinical relevance necessary for effective implementation.

## 5 Discussion

Understanding how important it is for all health professionals from all medical disciplines to be involved in research (and what happens when they are not) helps us see how we can encourage their participation. To accomplish this goal, the government and society need to create a strong supporting system, build a culture that values clinical research, and find ways to include it in our day-to-day practice. This will not only benefit the doctors but ultimately improve patient care as well (1, 14). Here are some key strategies identified to encourage clinician participation in research:

### 5.1 Institutional support and infrastructure

Healthcare institutions should emphasize research through the availability of resources, funds, and infrastructure for conducting research. The creation of research departments, access to research facilities, and various forms of financial incentives may encourage physicians to take up research activities. Likewise, infusing a research-friendly culture that recognizes the scientific contributions of health professionals can promote the research orientation of healthcare institutions.

### 5.2 Research training and education

Integrating research training into medical education and residency programs would give physicians and health professionals essential skills and knowledge for conducting high-quality research. That is why continuing medical education (**CME**) is important for research methodologies, data analysis, and evidence-based practices, as well as the value of mentorship and collaboration with experienced researchers.

### 5.3 Collaborative research

Interdisciplinary and collaborative research is vital for expanding our knowledge and discovering innovative solutions to face complex medical challenges. Therefore, partnerships between academia, healthcare, and industry should also be stimulated to identify potential opportunities for joint projects. These efforts will definitively enhance the quality and impact of research, ultimately benefiting patient care and pushing forward medical science.

### 5.4 Recognition and rewards

Rewarding and recognition of research contributions go a long way in motivating physicians to undertake research activities. Healthcare institutions can institute awards, grants, and recognition programs to recognize excellence in research contributions. Opportunities for presenting research work at conferences, publishing in reputable journals, and academic promotions also encourage clinicians to participate in research activities.

### 5.5 Integration of research in clinical practice

Balancing clinical practice with research responsibilities will be essential to ensure that clinician burnout is avoided while maintaining the best quality of care for patients. A healthcare institution may thus allow flexibility in scheduling and offer protected research time and/or administrative support, which enable physicians to get involved in research. The discussions on workload management and work-life balance can promote an enabling environment for undertaking research together with their clinical obligations.

## 6 Conclusion

Physicians should take an active role in research if we want to advance medical knowledge, enhance patient care, and support social development. Their firsthand experiences from working with patients, when paired with research activities, spark new ideas for diagnosis, treatment, and prevention, which leads to better quality healthcare for everyone.
